# Trends in health resource disparities in primary health care institutions in Liaoning Province in Northeast China

**DOI:** 10.1186/s12939-018-0896-8

**Published:** 2018-12-04

**Authors:** Shuping Wang, Jin Xu, Xiaofeng Jiang, Chaofan Li, Hongmin Li, Suhang Song, Erdan Huang, Qingyue Meng

**Affiliations:** 1 School of Health Care Management, Shandong University, 44 Culture Road, Li Xia District, Jinan, 250012 Shandong Province China; 2 NHC Key Laboratory of Health Economics and Policy Research(Shandong University), No 44 Wenhua Rd, Mailbox 128, Jinan, 250012 Shandong China; 3 China Center for Health Development Studies, School of Public Health, Peking University, 38 Xueyuan Road Haidian district, Beijing, 100191 China; 4 China National Health Development Research Center, 38 Xueyuan Road Haidian district, Beijing, 100191 China; 5 School of Public Health, Peking University, 38 Xueyuan Road Haidian district, Beijing, 100191 China

**Keywords:** Disparity, Quantity, Quality, Health resources, Primary health care institutions

## Abstract

**Background:**

The allocation of health resources in primary health care institutions (PHCI) is crucial to health reform. China has recently implemented many reform measures emphasizing the provision of primary health care services, with equity as one of the major goals. The aim of this study was to analyze the quantity, quality, and distribution of health resources in Liaoning Province from 2005 to 2017.

**Methods:**

Data were drawn from the annual financial report from 2005 to 2017 and information from the Liaoning Province Department of Statistics. Numbers of beds and physicians were used as indicators of health resources. Capital assets per bed, value of medical equipment per bed, operational space per bed, and number of physicians with different educational levels were used as indicators of quality of health resources. Concentration indices (CI) and Gini coefficients were calculated.

**Results:**

There was a steady rise in health resources in PHCI. From 2005 to 2017, the quality of health resources improved. The CI of beds showed an overall downward trend, indicating an improvement in the disparity among PHCI. There was a similar trend in the CI of fixed assets per bed. The Gini coefficients of physicians overall and physicians with different educational levels were almost always < 0.3, showing preferred equity status. There was a decreasing trend in the Gini coefficients of PHCI physicians with bachelor’s degrees or higher and physicians with associate’s degrees. The proportion of health resource of PHCI in health system increased from 2005 to 2009, before decreasing from 2009 to 2017 and the percentage of physicians overall and physicians with bachelor’s degrees or higher in PHCI declined after 2011.

**Conclusions:**

There was an improvement in the quantity and quality of health resources in PHCI from 2005 to 2017. The distribution of health resource allocation in PHCI also improved. The findings revealed that the measures for the improvement of PHCI physicians’ educational level has been successful and the measures taken by the government in health reform to strengthen the primary health care system have not been successful.

**Electronic supplementary material:**

The online version of this article (10.1186/s12939-018-0896-8) contains supplementary material, which is available to authorized users.

## Background

Primary health care, as a basic health protection for people, is essential to the success and sustainability of health systems. In China, the primary healthcare system provides generalist clinical care and basic public health services. From ancient times to the present, primary health care has been considered the basis of a good health strategy [1]. Extensive reviews of the literature have shown that effective primary care is associated with improved access to health care services, better population health, reduced hospitalizations, more cost effectiveness, and enhanced equity [2, 3].

Health care access is recognized as a fundamental human right. The distribution of a health care delivery system is an important component of health care access. Equity is one of the basic principles of the allocation of health resources, and it is the basis for achieving fairness in the provision of health services [4]. Evidence indicates that access to primary health care can play a crucial role in promoting regional health equity [5–7]. The equitable allocation of health resources helps to deliver effective resources to those most in need and to ensure accessibility to basic health services and fairness for vulnerable populations [8]. Therefore, research on the fairness of health resource allocation in primary health care is of great significance for the realization of equity in basic health services [9].

Liaoning is a developed province in northeast China. In 2017, Liaoning ranked fourteenth (of 31 total provinces) for gross domestic product (GDP) per capita. Its permanent population as of 2017 was 43.89 million. Of this number, 67.37% are urban dwellers and the remaining 32.63% live in rural areas. Liaoning’s population is aging, with 13.22% of the population aged over 65 years in 2017, representing a substantial increase from 9.76% in 2005. Population aging will result in an increase in the incidence of non-communicable diseases and demand for health care [10–12]. The structure of health resources in Liaoning Province is problematic, with an imbalance among regions [13, 14] and between urban and rural areas [15]. Some studies have found that the quality of health workers in primary health care institutions (PHCI) was low and that the structure of health resources was unreasonable [16]. Therefore, from 2005 to 2017, the government took many measures to improve the primary care system and to enhance primary health care in the province.

A first aim was to strengthen primary health care by improving the imbalance in health resources through building and strengthening primary health care infrastructure, especially in rural areas. The government allocated significant funds to PHCI; for example, about 90 million renminbi (RMB) was invested in medical equipment for new township health centers [17], and 509 million RMB was used to rebuild or upgrade one to three township health centers for each county and 154 community health centers. Remote and poor areas, places with high numbers of ethnic minorities, and endemic areas were targeted as priorities for investment [18].

Second, the government took measures to increase quantity and improve quality in the health workforce in PHCI. From 2005 to 2017, the government recruited more than 10,000 physicians and registered nurses to meet the demand in township health centers. To improve the quality of health workers, the government provided an education promotion program and training programs for PHCI health personnel. For example, each year from 2008 to 2017, 4500 health technicians were selected from rural health institutions to receive academic education and earn a college or university diploma, with the provincial government covering all tuition costs. Until 2011, health technicians who already had associate’s degrees were able to graduate from universities to receive the bachelor’s degrees.

Some studies have explored inequity in resources and services within the primary care sector [19, 20], and those studies have shown that the new health reform in 2009 promoted improving distribution in the number of health resource in PHCI among different provinces or cities [21–25]. However, other studies have found that the investment in the new health reform did not seem to lead to a successful primary care system [26]. The recent reforms prioritize the development of good primary care based on the existing situation [27]. Existing studies have analyzed the quantity and distribution of health resources in PHCI, but they have not analyzed changes in quality of health resources in PHCI. Therefore, the purpose of this study was to compare changes in the quantity, quality, and distribution of health resources in PHCI over 12 years (2005–2017) in Liaoning Province. The results of this study would be helpful to reflect effect of measures to improve the health resources in PHCI taken by the government and can be references for the government to formulate health policies for strengthening the primary health care system.

## Methods

### Data sources

We collected data on the total population, GDP per capita, and health resources in PHCI in 14 cities in Liaoning Province. The population and GDP per capita data were taken from the *Liaoning Statistical Yearbook* from 2006 to 2018. The data on the quality of beds were taken from the annual financial reports on PHCI from 2005 to 2017. Other data (including the numbers of physicians with different educational levels) were taken from the Department of Statistics of Liaoning Province.

### Measurements of inequity

The concentration index (CI) and the Gini coefficient have been identified as superior tools for measuring inequity [28]. The CI is defined as twice the area between the concentration curve (cumulative proportion of resources/services mapped onto the corresponding cumulative proportion of wealth) and the line of equality: C = 2cov(x,h)/μ, where x is the fractional rank in terms of GDP per capita, h is the indicator for health resources and services, and μ is the mean of the health indicator. C ranges from − 1 to 1: A value of zero indicates absolute equity, a negative value indicates a concentration of health resources or services among the poorer populations, and a positive value indicates a concentration of health resources or services among the richer populations. Because of limitations in data availability, we did not use standardization in estimating CI.

The Gini coefficient examines the distribution of health resources and services against population status [29]. The Gini coefficient was calculated based on the Lorenz curve—a graphical representation of the function of the cumulative proportion of resources of ordered institutions mapped onto the corresponding cumulative proportion of their size. This reflects the ratio of the area of the Lorenz curve and the diagonal line to the whole area below the 45^。^line,

$$ {S}_1=\frac{1}{2}\sum \limits_{i={1}^{i=0}}\left({Y}_i+{Y}_{i+1}\right){X}_{i+1}G=2\times \left(0.5-{S}_1\right)\times {S}_1 $$

where S_1_ is the area bounded by the Lorenz curve, Y_i_ is the cumulative proportion of health resources (Y_0_ = 0), and X_i + 1_ is the cumulative proportion of each group of the population or geographical area. G ranges from 0 to 1; a value of 0 indicates equitable distribution of resources or services, a value of less than 0.3 shows preferred equity status, a value of greater than 0.4 triggers an alert of inequity, a value exceeding 0.6 reflects a highly inequitable state [30].

### Main indicators

McCollum’s [31] work has identified human resources for health, equipment, and facilities as constituting quality of primary care. Health personnel can be classified according to their educational background and qualification. In terms of educational level, health professionals can be divided into postgraduate or undergraduate (bachelor’s degree or higher), junior college (associate /vocational degree), [32] and other (technical school or lower) [33, 34]. In our study, “other” included health professionals with technical secondary school, high school, or lower educational levels. Because of the government’s investment in infrastructure and medical equipment of PHCI, we used capital assets per bed [35, 36], the value of medical equipment per bed [37], and the operational space per bed [36, 38] as indicators to assess the improvement in the quality of beds in PHCI.

The CI index was estimated with four indicators, including the number and quality of beds in PHCI, from 2005 to 2017. The Gini coefficient was estimated with four indicators gauging the number and quality of physicians in PHCI from 2005 to 2017. We analyzed the allocation of health resources from the perspective of population distribution. The specific definitions and criteria for health resources are described below.

Physicians included licensed physicians and assistant licensed physicians—those staff members with a “licensed physician” or “assistant licensed physicians” title on their medical practitioner certificate who worked in the field of medical prevention and health care. Those who worked in management were not included. Licensed physicians have bachelor’s degrees or higher and majored in medicine at colleges or universities. Assistant licensed physicians are graduates of junior colleges, colleges, or universities and hold medical vocational degrees.

Beds in health care institutions refer to the actual number of beds in these institutions, including formal beds, simple beds, care beds, and beds that are being disinfected or repaired. Excluded here were neonatal beds, pre-delivery beds, observation beds, temporary beds, and beds for patients’ accompanying family members.

In the present study, PHCI include urban community health centers (stations) and rural township health centers. These institutions are responsible for providing basic medical and public health services to community residents.

## Results

**Fig. 1:**
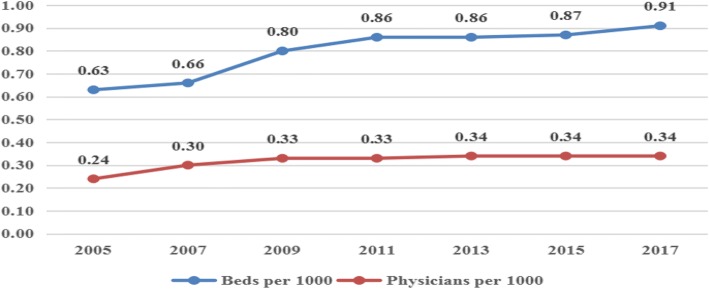
Time trends in the quantity of health resources in PHCI from 2005 to 2017

From 2005 to 2017, the number of beds per 1000 population increased from 0.63 to 0.91.The number of physicians in PHCI per 1000 population increased from 0.24 to 0.34. The specific values are shown in Fig. 1.

The fixed assets of medical facilities represent the capital of the medical service system, and medical equipment and operational space constitute the basic environment for supplying health services. The value of capital assets per bed in PHCI increased more than three-fold from 2005 to 2017. Financial investment in upgraded medical equipment in PHCI has gradually grown, and the value of medical equipment per bed increased yearly. The PHCI/hospital ratio of fixed assets per bed increased from 2005 to 2011 but decreased noticeably in 2017. The PHCI/hospital ratio of the value of medical equipment per bed was about 0.24–0.32, and this showed an increasing trend. The PHCI/hospital ratio of operational space per bed showed a similar trend as the ratio of fixed assets per bed. The specific values are shown in Table 1.

**Table 1:** Time trends in the quality of beds in PHCI from 2005 to 2017

Year	Capital assets per bed(10,000RMB)		Value of medical equipment per bed(10,000RMB)		Operational space per bed(m^2^)	
	PHCI	PHCI/hospital ratio	PHCI	PHCI/hospital ratio	PHCI	PHCI/hospital ratio
2005	3.30	0.28	1.28	0.24	43.24	0.81
2007	4.83	0.35	1.37	0.21	45.25	0.83
2009	5.64	0.37	1.78	0.24	43.60	0.86
2011	7.62	0.42	2.93	0.32	45.44	0.89
2013	8.50	0.33	3.63	0.32	46.39	0.83
2015	9.50	0.32	4.15	0.32	46.44	0.84
2017	10.92	0.32	4.98	0.32	46.56	0.84

**Fig. 2:**
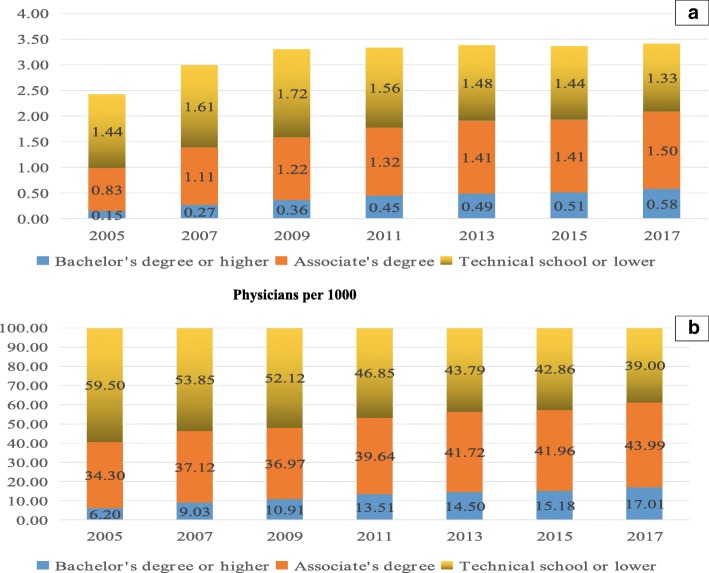
Time trends in the quality of physicians in PHCI from 2005 to 2017 The top panel presents the number of PHCI physicians with different degree types (Bachelor’s degree or higher,Associate’s degree, Technical school or lower) per 1000 population. The bottom panel represents the percentages of PHCI physicians with different degree types (Bachelor’s degree or higher, Associate’s degree, Technical school or lower).

For physicians’ education level, the number of physicians with bachelor’s degrees or higher per 1000 population exhibited an increasing trend from 2005 to 2017. The number of physicians with associate’s degrees per 1000 population showed a similar trend as that observed for physicians with bachelor’s degrees or above. Physicians with bachelor’s degrees or above accounted for 17.01% of all physicians in 2017, an increase from only 6.20% in 2005. The percentage of physicians with associate’s degrees also increased, from 34.30% in 2005 to 43.99% in 2017 (Fig. 2).

**Fig. 3:**
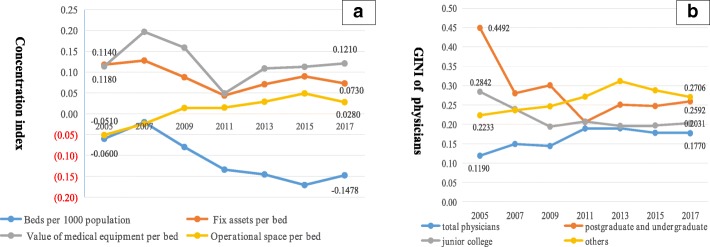
Distribution of health resources in PHCI .Panel **a** presents CI of the quantity and quality of beds in PHCI (beds per1000 population, fixed assets per bed,value of medical equipment per bed, operational space per bed).Panel **b** presents the Gini coefficients for the number of PHCI physicians by educational level (All physicians, postgraduate and undergraduate, junior college,or others) per 1000 population

The CI of value of medical equipment per bed in PHCI was high (ranging from 0.114 to 0.121). The CI value of fixed assets per bed in PHCI ranged from 0.118 to 0.073 and showed a decreasing trend. The CI of value of medical equipment and value of fixed assets per bed was a positive value, which indicated a concentration of the quality of beds among the richer populations. The CI value of operational space per bed increased slightly, ranging from − 0.051 in 2005 to 0.028 in 2017. The CI value of beds per PHCI was small, ranging from − 0.06 to − 0.148), which indicated a concentration of the quantity of beds towards the poorer populations and there was good wealth-related equality in this indicator (Fig. 3a and Additional file 1).

From 2005 to 2017, the Gini coefficients for the number of physicians per 1000 in PHCI ranged from 0.119 to 0.177 overall. This range was 0.4492–0.2592 for the number of physicians with bachelor’s degrees or higher, 0.2842–0.2031 for the number of physicians with associate’s degrees, and 0.2233–0.2706 for the number of physicians with other degrees (Fig. 3b and Additional file 2). The Gini coefficients for the numbers of physicians with bachelor’s degrees or higher and with associate’s degrees showed a decreasing trend, demonstrating an improvement in the distribution of high-quality physicians.

**Fig. 4:**
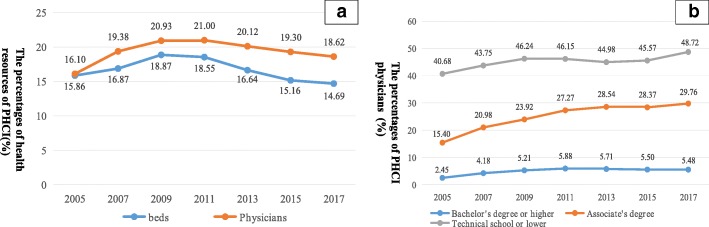
Percentages of health resources found in PHCI. Panel **a** presents the percentages of health resources (beds, physicians) in PHCI. Panel **b** presents the percentages of physicians with different types of degrees (bachelor’sdegree or higher, associate’s degree, or technical school or lower) in PHCI

Figure 4 depicts changes over time in the percentages of health resources in PHCI. The proportion of all physicians working in PHCI increased from 16.10% in 2005 to 20.93% in 2009, followed by a decrease to 18.62% in 2017. The percentage of beds in PHCI showed a decline, despite an increase from 15.86% in 2005 to 18.87% in 2009. The percentage of physicians with bachelor’s degrees or higher increased from 2005 to 2011, followed by a decrease until 2017. From 2005 to 2017, there was also an increase in the percentage of physicians with associate’s degrees (Fig. 4). From 2005 to 2009, the percentage of physicians in PHCI increased by 4.83%, and the proportion of beds in PHCI increased by 3.01%. From 2009 to 2015, the percentage of physicians in PHCI decreased by 2.31%, and the percentage of beds in PHCI decreased by 4.18% (Fig. 4).

## Discussion

This study analyzed trends in the quantity and quality of beds and physicians in PHCI, finding an increasing trend in the number of beds and physicians. However, there was a decreasing trend in the percentages of beds and physicians in PHCI after the health reform, after these percentages generally increased from 2005 to 2009. There was a trend toward improvement in the quality of beds and physicians and in disparities in the quality of beds and physicians across different cities.

First, the study found a steady rise in the number of health resources in PHCI. This result was similar to the findings of Zhang [25] and Xu [24]. On the demand side, expanding health insurance coverage in Liaoning Province may explain this result. Together, the NCMS (New Cooperative Medical System), the urban-based basic medical insurance scheme, and the Urban Employee Basic Medical Insurance program covered more than 99% of the population in 2017, representing an increase from 40.1% of the population in 2005. The hospitalization rate for NCMS enrollees increased from 0.95 to 11.78% from 2005 to 2017 in Liaoning Province. Other previous studies [39, 40] have reported similar results, indicating that the introduction of the NCMS has increased the use of inpatient and outpatient health services. On the supply side, an increase in the number of urban community health centers caused an increase in the numbers of beds and physicians from 2005 to 2017. After the health reform, the government recruited more than 10,000 physicians and registered nurses to meet the demand for township health centers.

Second, the present study found improvements in the quality of beds and physicians. From 2005 to 2017, the government made financial investments in infrastructure and updated medical equipment in PHCI. In a previous study, many interviewees in PHCI described medical equipment in PHCI as important for gaining the trust of residents and for retaining staff [41]. Thus, improvements of the quality of health resources may improve the environment in PHCI. The government also sponsored a free education upgrade program for health professionals in PHCI. This program might have driven the increase in the numbers of health professionals in PHCI with bachelor’s degrees or higher and with associate’s degrees. All of these measures, including capital assets per bed, value of medical equipment per bed and the number of physicians with bachelor’s degrees or higher, indicate the improvement of the quality of health resources.

Third, the CI of beds showed an overall downward trend, indicating an improvement in the disparity in PHCI beds across different cities—a result also found by Liu [42]. There was a similar trend in the CI of fixed assets per bed. One reason for this was that the government investment in infrastructure and medical equipment came from central and provincial governments and prioritized investment in lower-income regions. The Gini coefficients for physicians overall and physicians with different educational levels were less than 0.3, except for physicians with bachelor’s degrees or higher in 2005, which showed preferred equity status. The Gini index for PHCI physicians with bachelor’s degrees or higher and for physicians with associate’s degrees decreased from 2005 to 2017, indicating a continuous improvement in the equity of the health workforce allocation. This result was consistent with the findings of other studies [43–45]. Free adult education for health professionals in PHCI with bachelor’s or associate’s degrees gave priority to participants from lower-income areas, ethnic minorities, and those working in resource-poor areas. Admission score for those participants added 20 points. Another reason for this finding is the salary reform. Many health workers in wealthier areas found that the salary reform reduced their income and this may promote “brain drain” among health professionals in PHCI. This finding was similar to the results of a previous study [46].

Fourth, the present study’s findings regarding the percentages of health resources in PHCI indicated the role of the primary health care system. We observed declining trends in the percentages of beds and physicians found in PHCI after the health reform, followed by an increase from 2005 to 2009. This result was similar to findings reported by Wu [47] and Zhang [48]. On the demand side, one reason for this finding may be that the implementation of the universal medical insurance system contributed to an increase in the numbers of physicians and beds in the health system [49, 50]. Another explanation for the finding is that patients do not trust PHCI because of shortages of primary care practitioners and medical facilities [1, 51], which has led to increasing numbers of urban residents preferring to go to large hospital centers, irrespective of the nature of the disease they have. One explanation for this finding on the supply side may be the establishment of an essential medicines program for PHCI; the low prices of some drugs have eroded profits to such an extent that drug companies are unwilling to produce and deliver them [52, 53]. This has led to the lack of availability of some drugs at township level. This situation has driven patients to the more expensive county hospitals, leaving beds and equipment in PHCI unused [39]. Another explanation for this finding was salary reforms for PHCI, which introduced fixed salaries for township health workers set by the local government. These reforms led to the loss of income from drug sales, leaving health workers with salaries equivalent to the salaries of secondary school teachers. The salary reforms have contributed to the challenge of recruiting and retaining rural doctors, jeopardizing the primary reform goal of strengthening primary care [46]. The lack of medicines and these salary reforms caused many PHCI to stop providing inpatient services, which caused health technicians to leave to work at larger hospitals. This result is similar to the results of research conducted by Fu [54] and Xu [46]. A final explanation for this finding was that hospitals and PHCI competed for patients. Hospitals tended to expand their scale and services to attract more patients and more profit, so more resources have been poured into hospitals, further exacerbating the disparities between hospitals and PHCI [55].

The present study had several limitations. First, the disaggregated data used in this manuscript can only reflect the health resource allocation status at the cut-off point of this work. It was not possible to provide a complete reflection of the whole picture. Second, although some studies have found that village doctors serve as the backbone of the medical system, providing basic medical care and public health services, we were unable to examine village clinics’ role in the primary health care system because of the lack of the data on village doctors and their educational level. Third, in this study, we chose indicators for the quality of health resources rather than indicators of the quality of health services. Interview results in previous work suggest that training programs for health technicians have improved health workers’ abilities, but this situation could not be shown completely in the present study. Finally, many measures for strengthening PHCI have been implemented from 2005 to 2017, and future research should assess the effect of each of these measures on the distribution of health resources.

## Conclusions

Based on the analysis above, we find that the quantity and quality of health resources in PHCI improved in Liaoning Province from 2005 to 2017. According to the analysis of CI and the Gini index, the distribution of health resources improved gradually. The findings revealed that the measures for the improvement of PHCI physicians’ educational level has been successful. However, the proportions of total beds and physicians found in PHCI decreased after the health reform, demonstrating that the measures taken by the government to strengthen the primary health care system were not successful. Therefore, to successfully strengthen the primary health care system, policies should not pay attention only to medical equipment, facilities, and the health workforce; they should also focus on the “software,” such as skills, teamwork, operational model, and a culture of cooperation among staff members.

## Additional files

Additional file 1: The trends of CI of quantity and quality of beds in PHCI from 2005 to 2017 (DOC 202 kb) (DOCX 14 kb)

Additional file 2: The trends of GINI coefficients of quantity and quality of physicians in PHCI (DOCX 14 kb)

## Abbreviations

CI: Concentration Index
GDP: Gross Domestic Product
NCMS: New Cooperative Medical System
PHCI: Primary Health Care Institutions
RMB: Renminbi
