# Supplementary material for: Trends in health resource disparities in primary health care institutions in Liaoning Province in Northeast China
Source: Int J Equity Health. 2018 Dec 4;17:178. doi: 10.1186/s12939-018-0896-8 (PMC6280446; doi:10.1186/s12939-018-0896-8)
Supplement: Supplementary file 1 — The trends of CI of quantity and quality of beds in PHCI from 2005 to 2017 (DOC 202 kb) (DOCX 14 kb) [file 12939_2018_896_MOESM1_ESM.docx]

Additional file 1 The trends of CI of quantity and quality of beds in PHCI from 2005 to 2017

| Year | Beds per1000 population | Fix assets per bed | Value of medical equipment per bed | Operational space per bed |
| --- | --- | --- | --- | --- |
| 2005 | -0.0600 | 0.1180 | 0.1140 | -0.0510 |
| 2007 | -0.0202 | 0.1280 | 0.1970 | -0.0240 |
| 2009 | -0.0797 | 0.0880 | 0.1590 | 0.0140 |
| 2011 | -0.1341 | 0.0440 | 0.0490 | 0.0150 |
| 2013 | -0.1457 | 0.0710 | 0.1090 | 0.0290 |
| 2015 | -0.1711 | 0.0900 | 0.1130 | 0.0490 |
| 2017 | -0.1478 | 0.0730 | 0.1210 | 0.0280 |
