# Supplementary material for: Trends in health resource disparities in primary health care institutions in Liaoning Province in Northeast China
Source: Int J Equity Health. 2018 Dec 4;17:178. doi: 10.1186/s12939-018-0896-8 (PMC6280446; doi:10.1186/s12939-018-0896-8)
Supplement: Supplementary file 2 — The trends of GINI coefficients of quantity and quality of physicians in PHCI (DOCX 14 kb) [file 12939_2018_896_MOESM2_ESM.docx]

Additional file 2 The trends of GINI coefficients of quantity and quality of physicians in PHCI

| Year | Total physicians | Postgraduate and undergraduate | Junior college | Others |
| --- | --- | --- | --- | --- |
| 2005 | 0.1190 | 0.4492 | 0.2842 | 0.2233 |
| 2007 | 0.1492 | 0.2802 | 0.2394 | 0.2364 |
| 2009 | 0.1440 | 0.3008 | 0.1941 | 0.2465 |
| 2011 | 0.1892 | 0.2061 | 0.2071 | 0.2715 |
| 2013 | 0.1898 | 0.2507 | 0.1960 | 0.3116 |
| 2015 | 0.1781 | 0.2473 | 0.1972 | 0.2879 |
| 2017 | 0.1770 | 0.2592 | 0.2031 | 0.2706 |
